# Anti-type II collagen antibodies, anti-CCP, IgA RF and IgM RF are associated with joint damage, assessed eight years after onset of juvenile idiopathic arthritis (JIA)

**DOI:** 10.1186/1546-0096-12-22

**Published:** 2014-06-11

**Authors:** Lillemor Berntson, Ellen Nordal, Anders Fasth, Kristiina Aalto, Troels Herlin, Susan Nielsen, Marite Rygg, Marek Zak, Johan Rönnelid

**Affiliations:** 1 Department of Women’s and Children’s Health, Uppsala University, Uppsala, Sweden; 2 Department of Pediatrics, University Hospital of North Norway, Tromsø, Norway; 3 Institute of Clinical Medicine, University of Tromsø, Tromsø, Norway; 4 Department of Pediatrics, University of Gothenburg, Gothenburg, Sweden; 5 Department of Pediatrics, Children’s Hospital, Helsinki University Hospital, Helsinki, Finland; 6 Århus University Hospital, Skejby, Århus, Denmark; 7 University Clinic of Pediatrics II, Rigshospitalet, Copenhagen, Denmark; 8 Department of Laboratory Medicine, Children’s and Women’s Health, Norwegian University of Science and Technology, Trondheim, Norway; 9 Department of Pediatrics, St. Olavs Hospital, Trondheim, Norway; 10 Department of Immunology, Genetics and Pathology, Uppsala University, Uppsala, Sweden

**Keywords:** Arthritis, Juvenile Idiopathic Arthritis, Anti-CCP, Rheumatoid factor, Anti-collagen type II antibodies, Child, Joints

## Abstract

**Background:**

Early appearance of antibodies specific for native human type II collagen (anti-CII) characterizes an early inflammatory and destructive phenotype in adults with rheumatoid arthritis (RA). The objective of this study was to investigate the occurrence of anti-CII, IgM RF, IgA RF and anti-CCP in serum samples obtained early after diagnosis, and to relate the occurrence of autoantibodies to outcome after eight years of disease in children with juvenile idiopathic arthritis (JIA).

**Methods:**

The Nordic JIA database prospectively included JIA patients followed for eight years with data on remission and joint damage. From this database, serum samples collected from 192 patients, at a median of four months after disease onset, were analysed for IgG anti-CII, IgM RF, IgA RF and IgG anti-CCP. Joint damage was assessed based on Juvenile Arthritis Damage Index for Articular damage (JADI-A), a validated clinical instrument for joint damage.

**Results:**

Elevated serum levels of anti-CII occurred in 3.1%, IgM RF in 3.6%, IgA RF in 3.1% and anti-CCP in 2.6% of the patients. Occurrence of RF and anti-CCP did to some extent overlap, but rarely with anti-CII. The polyarticular and oligoarticular extended categories were overrepresented in patients with two or more autoantibodies. Anti-CII occurred in younger children, usually without overlap with the other autoantibodies and was associated with high levels of C-reactive protein (CRP) early in the disease course. All four autoantibodies were significantly associated with joint damage, but not with active disease at the eight-year follow up.

**Conclusions:**

Anti-CII, anti-CCP, IgA RF and IgM RF detected early in the disease course predicted joint damage when assessed after eight years of disease. The role of anti-CII in JIA should be further studied.

## Background

Juvenile idiopathic arthritis (JIA) is a heterogeneous condition with time of onset before the age of 16. The diversity in clinical manifestations differs from that in adult arthritis, but some findings point to similarities among children and adults. Reliable biomarkers for prediction of outcome and choice of treatment in JIA are scarce [1].

JIA is currently described by the International League of Associations for Rheumatism (ILAR) classification system as a disease comprising seven categories, based on clinical characteristics, heredity and laboratory results [2]. While IgM rheumatoid factor (RF) is a determinant of the polyarticular categories, occurrence of IgA RF and anti-citrullinated peptide antibodies (ACPA), for which the most common clinical test is anti-cyclic citrullinated peptide (anti-CCP), are not included in the ILAR classification.

In population-based studies on JIA, IgM RF has an occurrence rate of 2–3% [3,4] and defines a subset of JIA resembling that of rheumatoid arthritis (RA) with a polyarticular disease, higher age at onset and a higher risk of joint damage. IgA RF has been studied far less, but is also discussed as a risk factor for joint space narrowing or joint erosions [5].

In RA, anti-CCP can be detected several years before disease onset [6], has a high disease specificity, and is now included in the 2010 classification criteria for RA [7]. The prognostic role of anti-CCP in JIA resembles that of IgM RF. Anti-CCP has low prevalence (2–15%) and is present in particular in the RF-positive, polyarticular JIA category [8-10]. The majority of JIA study cohorts on ACPA are small. Results depend on the methods used for analysis, cut-off values for autoantibody analyses and patient selection.

Autoantibodies against collagen type II (anti-CII) have been studied in adult RA (11–14). CII is the predominant hyaline cartilage collagen. Patients with anti-CII make up a distinct RA phenotype, found in a minority of adult RA patients, associated with acute inflammation at disease onset [11] and early radiographic destruction [12]. Anti-CII has been shown to induce the proinflammatory cytokines TNFα, IL-1β and IL-8, when incorporated in immune complexes *in vitro*[13]. These findings are in concordance with earlier reports showing that high anti-CII levels are associated with higher levels of ESR, CRP, TNFα and IL-6 compared with what is found in anti-CII negative RA patients [14]. Anti-CII positive RA patients also experience less diagnostic delay [11], probably because of the high inflammatory activity. In contrast to ACPA and RF, anti-CII does not precede the development of RA by a long time period [6,15]. Earlier studies have shown that levels of anti-CII decrease shortly after RA diagnosis [11,16], and therefore studies of anti-CII should preferably be performed on sera obtained early at or soon after disease onset. The anti-CII phenotype in adult RA thus seems to be a temporary finding around the time of symptom onset and diagnosis [12]. Data regarding anti-CII in JIA are scarce [17].

Our aim was to study whether early occurrence of anti-CII, IgM RF, IgA RF and/or anti-CCP, in a well-defined cohort of children with JIA, could be related to outcome data and joint damage after eight years of disease.

## Methods

### Patients

The Nordic JIA database consists of 440 patients followed prospectively for a median of 97 months (IQR 95–105) in a population-based approach and classified according to the ILAR criteria after eight years of disease [2]. From this database, 192 patients with (available) sera taken within 12 months after disease onset were included. Due to these inclusion criteria, this sub-study cannot be considered population-based. Data of CRP results ≤ 10 months after disease onset were included if available. At the eight-year follow-up visit, remission according to the preliminary criteria of Wallace et al. [18], and joint damage according to Juvenile Arthritis Damage Index for Articular damage (JADI-A) (scale 0–72 where 0 means no damage) [19], were assessed by a paediatric rheumatologist in 147 of the patients. We found no statistical difference between the original 440 patients and the 147 patients studied as regards gender, age of onset or proportion of oligoarticular persistent patients.

### Analyses of autoantibodies

For autoantibody analyses we used sera stored at −70°C, collected within a median of four months (IQR 2–7) after disease onset. IgM RF, IgA RF and anti-CCP were analysed using the Phadia ImmunoCAP enzyme immunoassay, allowing isotype-specific analysis of IgM and IgA RF. For anti-CCP, the cut-off value at Uppsala University Hospital of > 7 U/ml was used, which is a commonly used cut-off value in many laboratories. The reference ranges for IgM (>5 U/ml) and IgA RF (>9 U/ml) were established with 100 healthy adult donors, using the reference range criteria (>95% diagnostic specificity) determined based on the 1987 ACR RA classification criteria [20]. Analysis of anti-CII IgG was performed with an in-house enzyme-linked immunosorbent assay (ELISA), using the cut-off (>29 U/ml) set at 95% specificity among 100 healthy adult blood donors as described earlier [11,12].

### Statistical methods

Conventional descriptive statistics were used; median and interquartile ranges (IQR) were given. For independent samples the Mann–Whitney U test was used and for analysis of proportions Fisher’s exact test was employed, due to the low number of antibody-positive patients. Correlations were assessed using Spearman’s rank correlation coefficients (ρ). P-values of less than 0.05 were considered statistically significant for two-tailed tests. Analyses were carried out using the Statistical Package for Social Sciences (SPSS), version 20 (IBM SPSS Statistics) and JMP (SAS Institute).

### Ethical issues

The Research Ethical Committees in each country gave their approval in accordance with national practice and legislation. In Sweden the study was approved by the Regional Ethical Review Board at Uppsala. Written informed consent was obtained from patients > 16 years and from parents for children aged < 16 years.

## Results

All ILAR categories were represented in the patient cohort, of which 69.3% were female (Table 1). The oligoarticular persistent group was the largest (46.9% of the total cohort). Elevated levels of anti-CII were found in 3.1% of the serum samples, IgM RF in 3.6%, IgA RF in 3.1%, anti-CCP in 2.6%.

**Table 1:** Prevalence of autoantibodies during the first year of disease in 192 patients from the Nordic juvenile idiopathic arthritis database classified after a median of eight years of disease according to the ILAR* criteria

ILAR* category	**Total cohort n (%)**	**Girls/boys n (% girls)**	**IgM RF- positive n (%)**	**IgA RF-positive n (%)**	**IgG anti-CCP positive n (%)**	**IgG anti-collagen II positive n (%)**
Systemic	9 (4.7%)	5/4 (55%)	1	0	1	0
Oligoarticular persistent	90 (46.9%)	60/30 (66.6%)	2	-	-	2
Oligoarticular extended	8 (4.2%)	8/0 (100%)	1	1	1	-
Polyarticular RF-negative	40 (20.8%)	30/10 (75.0%)	-	1	1	2
Polyarticular RF-positive	1	1/0	1	1	-	-
Juvenile psoriatic arthritis	1	0/1	-	-	-	-
Enthesitis-related arthritis	15 (7.8%)	5/10 (33.3%)	-	2	1	1
Undifferentiated arthritis	28 (14.6%)	24/4 (85.7%)	2	1	1	1
Total	192	133/59 (69.3%)	7 (3.6%)	6 (3.1%)	5 (2.6%)	6 (3.1%)

*International League of Associations for Rheumatology classification criteria [2].

Occurrence of RF and anti-CCP did overlap to some extent, but rarely with anti-CII (Table 2). The polyarticular and oligoarticular extended categories were overrepresented in patients with two or more of the four autoantibodies present. In two patients (patients 8 and 9 in Table 2), the anti-CCP value was more than ten times higher than in other anti-CCP positive patients.

**Table 2:** Patterns of autoantibodies collected in 17 of 192 patients from the Nordic juvenile idiopathic arthritis database

**Patient No**	**Age at time of onset**	**IgM RF Cut-off**	**IgA RF Cut-off**	**IgG Anti-CCP Cut-off**	**IgG Anti-Collagen II Cut-off**	**Articular damage**^**¤**^	**JIA category**^**#**^	**Cumulative number of joints affected during the first eight years of disease**
	**Years**	**> 5 U/ml**	**> 9 U/ml**	**> 7 U/ml**	**> 29 U/ml**			
1	4.4	5.7	-	-	-	Missing	Systemic	4
2	12.8	6.0	-	-	-	-	Oligoarticular persistent	2
3	13.5	23.0	-	-	-	-	Undifferentiated^*^	1
4	9.4	5.5	-	-	-	-	Oligoarticular persistent	2
5	9.3	-	13.0	-	-	-	Enthesitis-related arthritis	4
6	1.5	-	14.0	-	-	1	Oligoarticular extended	18
7	13.2	64.0	17.0	-	-	1	Polyarticular RF-positive	13
8	13.3	132.0	67.0	≥ 340	-	1	Undifferentiated^**^	18
9	10.9	17.0	-	≥ 340	-	1	Oligoarticular extended	24
10	1.1	-	-	9.8	-	-	Systemic	6
11	11.8	-	23.0	15.0	-	-	Enthesitis-related arthritis	2
12	11.0	-	11.0	12.0	94.3	1	Polyarticular RF-negative	19
13	11.7	-	-	-	35.9	1	Polyarticular RF-negative	34
14	4.0	-	-	-	46.6	-	Enthesitis-related arthritis	13
15	4.8	-	-	-	30.4	-	Oligoarticular extended	9
16	4.3	-	-	-	72.9	-	Undifferentiated^***^	2
17	8.1	-	-	-	230.8	Missing	Oligoarticular persistent	1

^¤^Articular damage at eight-year follow-up according to JADI-A (score 0–72) [19].

^#^ILAR Edmonton criteria after eight years of disease [2].

^*^Oligoarticular persistent joint pattern and presence of IgM rheumatoid factor on two occasions more than three months apart.

^**^Polyarticular pattern from onset and psoriasis in first degree relative.

^***^Oligoarticular persistent joint pattern and psoriasis in first degree relative.

The median age of onset was significantly higher in patients with IgM as compared with patients without these antibodies, while patients with anti-CII had onset ages comparable to those of the total JIA cohort (Table 3). JADI-A for joint damage was assessed in 147 patients and found to be > 0 in 18 patients. All four autoantibodies were significantly associated with joint damage according to JADI-A after eight years, with the strongest associations found for IgA RF and anti-CCP (Table 3). The occurrence of any of the investigated autoantibodies was also strongly associated with elevated JADI-A (p = 0.0004, Table 3). There was, however, no significant association between the presence of these autoantibodies and cumulative number of joints affected, ongoing disease activity, or remission at eight years.

**Table 3:** Analyses of autoantibodies in 147 patients from the Nordic JIA database followed for eight years in association with age at disease onset, number of cumulative joints, remission and articular damage

	**Total**	**Age at onset (n = 147)**		**Cumulative joints during the first eight years of disease (n = 147)**		**Remission after 8 years (n = 147)**		Articular damage*** at eight year follow up (n = 147)	
	**n (%)**	**Median (IQR)**	p*	**Median (IQR)**	p*	**Number of patients without remission/total number (%)**	p**	**Number of patients with damage/total number (%)**	p**
Total cohort	147	5.4 (2.8-9.9)		8 (3–15)		99/147 (67%)		18/147 (12)	
IgM RF positive	6 (4.1%)	10.5 (13.0-13.4)	0.002	8 (2–20)	0.79	3/6 (50%)	0.39	3/6 (50)	0.0068
IgA RF positive	6 (4.1%)	11.4 (7.6-13.2)	0.07	16 (4–18)	0.57	1/6 (17%)	0.66	4/6 (67)	0.0001
Anti-CCP positive	5 (3.4%)	11.0 (6.0-12.6)	0.15	18 (4–22)	0.89	1/5 (20%)	1.00	3/5 (60)	0.0007
Anti-collagen II positive	5 (3.4%)	4.8 (4.2-11.4)	0.54	13 (6–26)	0.29	2/5 (40%)	0.66	2/5 (40)	0.0274
Any conventional# antibody								5/11	0.0004
Any antibody¤								6/15	0.0004

*Mann–Whitney U test, as compared with patients without the corresponding autoantibody.

**Fisher’s Exact Test, as compared with patients without the corresponding autoantibody.

***Articular damage at eight-year follow-up according to JADI-A (score 0–72).

#Positive for one or more antibodies: IgM RF, IgA RF and/or anti-CCP.

¤Positive for one or more antibodies: IgM RF, IgA RF, anti-CCP and/or anti-collagen II.

**Figure 1:**
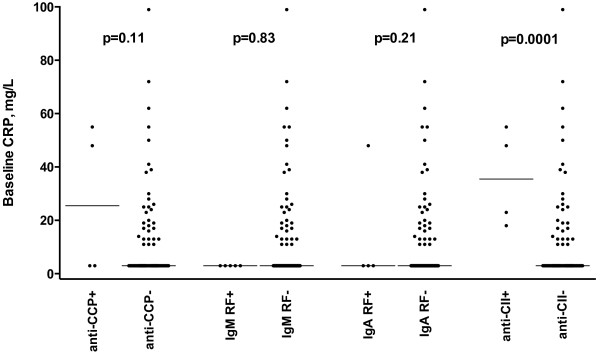
**Association between the occurrence of four autoantibodies and early CRP levels in 134 (of 192) patients with JIA where blood samples were obtained ≤ 10 months after onset of disease.** P-values denote differences in median CRP levels between patients with and without the corresponding autoantibodies investigated with the Mann-Whitney’s U test. Horizontal lines indicate median levels. All CRP levels ≤ 10 mg/L were set to 3 g/L.

When patient antibodies were correlated to the JADI-A scores, we found a highly significant association to the number of antibodies detected (p = 0.0001). The correlation coefficients were, however, low for the commonly tested antibodies (anti-CCP, IgM RF and IgA RF) (Spearman’s ρ = 0.30) and did not increase when anti-CII were included (Spearman’s ρ = 0.31).Information on CRP levels taken ≤ 10 months after disease onset were available for 134/192 patients. CRP taken early in the disease course were significantly higher (more than four times higher) in anti-CII positive patients than in anti-CII negative patients (median 30.0 vs. 8.7 mg/l; p = 0.0001). No such association was found for any of the other antibodies studied (Figure 1).

## Discussion

As far as we know, this is the first study investigating the long-term prognostic impact of antibodies against native CII in JIA. Occurrence of anti-CII rarely overlapped with anti-CCP, IgA RF and IgM RF, and was seen in a younger age group at disease onset than the other three conventional autoantibodies. All four autoantibodies were associated with clinical assessment of joint damage according to JADI-A after a median of eight years of disease.

A strength of this study is that it includes all JIA categories, and that analysed serum samples were collected early in the disease course. This is in contrast to other studies on autoantibodies in JIA, where samples have been collected at different times after disease onset. This early sampling was also a prerequisite for the analysis of anti-CII, as previous studies have shown that anti-CII levels peak around the time of RA diagnosis [11].

Limitations of our study were the small number of autoantibody-positive patients, and that no data on radiological damage was available for assessment of joint damage. The JADI-A index used in this study is validated and considered useful as an instrument for the clinical assessment of long-term articular damage in patients with JIA, but the index mainly collects severe articular changes [19]. Radiographical methods do not fully reflect the spectrum of articular damage in children since contractures and restricted range of movement can also depend on extraarticular damage. An advantage of using the JADI-A is that it includes restricted range of movement and joint damage not necessarily detected by radiography. A limitation is that autoantibodies were analysed at only one time point, which might include false positive serum samples. In order to be classified as RF-positive JIA, two positive tests at least three months apart are required (2). Adult and not age-matched cut-off values were used for the conventionally used autoantibodies (IgM RF, IgA RF and anti-CCP) [17]. We therefore decided to use adult cut-off levels also for anti-CII. All cut-off values were locally validated using 100 healthy individuals, and specificities adjusted to 95–97% according to the currently used definition of RF specificity as defined in the previous ACR classification criteria for RA [20], not changed in the more recent EULAR/ACR criteria [7].

Occurrence of autoantibodies was rare in our study, much like in the majority of previous studies on JIA [9,21-23]. As in adult RA, occurrence of IgM RF in JIA has been shown to correlate with joint damage or joint space narrowing [5,17,22,24-26]. Our results were in line with these studies, as we found a significant association with joint damage in the IgM RF-positive patients.

Children with oligoarticular JIA have occasionally been found to be IgM RF-positive. Our results gave no indications of a more severe disease in these children. The three IgM RF-positive patients in our study with high age at onset and oligoarticular joint pattern throughout the disease course did not have any joint damage after eight years of disease. These patients also lacked any of the other autoantibodies investigated in the study. This might indicate that positive IgM RF in an oligoarticular disease is of little significance if the patient lacks anti-CCP or IgA RF.

Several studies on JIA point to a high co-occurrence of IgM RF and anti-CCP [8,26,27], but they may also occur independently [28]. JIA patients with IgM RF and/or anti-CCP generally present at a higher age than patients without these antibodies [29]. In line with these results, the IgM RF-positive children with articular damage in our study had a high age at onset, a polyarticular or oligoarticular extended disease pattern, and were IgA RF or anti-CCP positive as well. The anti-CCP levels in these children were also very high. It has been suggested that JIA patients positive for both IgM RF and anti-CCP present a more severe disease phenotype than those with only one autoantibody [30]. In line with this, we can report that co-occurrence of IgA RF and possibly of anti-CII is associated with severe joint damage after eight years of disease in JIA.

We found an association between anti-CCP and joint damage. This is supported by several earlier studies showing an increased risk for radiological bone damage in patients with anti-CCP in JIA [17,22,26,28]. Four of five anti-CCP positive patients in our study were more than 10 years old at onset; the fifth was one year old and had systemic JIA. However, others have presented a separate group of children with only anti-CCP and young age of onset [21].

Two of the five anti-CCP positive patients had levels more than ten times higher than the rest of the group. This is in concordance with adult RA, where anti-CCP positive individuals generally have levels clearly above the cut-off [31]. Others have found anti-CCP levels to be higher in IgM RF-positive patients with JIA compared with IgM RF-negative patients [21]. A very high level of anti-CCP at diagnosis should most likely be considered in prognostic evaluation and risk assessment for articular damage in children with JIA [22].

Studies on IgA RF and joint damage in JIA are limited. In our study, we found the strongest association between IgA RF and joint damage after eight years of disease. This is supported by two previous studies presenting an increased risk for joint space narrowing or erosions in JIA patients with IgA RF [5,17]. In adults, a number of studies have indicated IgA RF as a stronger predictor for poor radiological prognosis than IgM RF [32,33]. High levels of IgA RF have also been shown to predict poor response to TNF inhibition in RA [34].

Only six patients were positive for anti-CII antibodies in our study, but the association with joint damage was statistically significant. Interestingly, this group of children had a median age of onset of 6.4 years, very close to that of the total cohort, in contrast to the IgM RF and anti-CCP positive patients, who had a higher age of onset (Table 3). The young age at onset and the low frequency of overlap with the other autoantibodies indicate that the anti-CII JIA phenotype might differ from the RF/anti-CCP positive phenotypes. In RA, elevated levels of anti-CII at diagnosis represent an acute onset phenotype with high CRP [11,12], whereas anti-CCP and RF-positive individuals do not differ from autoantibody-negative individuals at baseline, but have a significantly poorer prognosis [35]. Anti-CII and anti-CCP do not only represent clinically opposite phenotypes in adult RA, but their occurrence is statistically inversely related [11]. Our result of an increased risk of joint damage after eight years of disease in anti-CII positive JIA patient differs from findings in adult RA, where anti-CII positive individuals have acute inflammation [11] and more radiological destructions [12] at disease onset, but not later, when levels of anti-CII decline. Although the number of anti-CII positive JIA patients is low, our study indicates that CRP levels are high during early disease in anti-CII positive children with JIA, as they are in adults with RA. In the present study, the median early CRP level was more than four times higher among anti-CII positive than among anti-CII negative JIA patients. In a previous study, using exactly the same anti-CII ELISA as employed in this study, the mean CRP levels were increased 2.5-fold at the time of diagnosis in anti-CII positive adult RA patients (53 vs 21 mg/l, p < 0.0001) [11]. We hypothesize that these elevated CRP levels in anti-CII positive JIA patients may be due to production of proinflammatory cytokines by monocytes stimulated with anti-CII containing immune complexes, as was described in adult RA [11,13].

We do not know how anti-CII develops over time in JIA patients. In adult RA, levels are not increased before diagnosis [15] and quickly decrease after [11,16]. If anti-CII levels are only transiently elevated at the time of diagnosis in JIA, early serum sampling as employed in our study should be recommended.

We are only aware of one recent study on anti-CII in JIA [17]. In that study, encompassing 95 patients with disease duration varying between 0.4 and 9.5 (median 2.0) years and thus with later serum sampling than in our study, 17.9% had antibodies against native CII [11]. Anti-CII were not associated with more joint erosions at the time of serum sampling, but the study did not include any clinical or radiological follow-up data to evaluate the prognostic impact of anti-CII [17].

Our findings suggest that occurrence of anti-CII, anti-CCP, IgM RF or IgA RF analysed at an early stage of disease may predict later joint damage. We suggest analysis of IgM and IgA RF as well as anti-CCP in all newly diagnosed children with JIA. We also suggest special attention to patients with more than one of these autoantibodies present. The role for anti-CII in JIA should be more extensively studied.

## Conclusions

Occurrence of anti-CII, anti-CCP, IgM RF or IgA RF analysed at an early stage of disease may predict later joint damage. Patients with more than one of these autoantibodies present merit special attention. Anti-CII in JIA seems to characterize a different subset of JIA patients than the other antibodies, associated with increased CRP levels early after disease onset, and with clinical joint damage after eight years.

## Competing interests

The authors declare that they have no competing interests.

## Authors’ contributions

All authors were involved in drafting the article or revising it critically for important intellectual content. All authors also approved the final version to be published and agreed to be accountable for all aspects of the work. Dr. LB had full access to all data in the study and takes responsibility for the integrity of the data and the accuracy of the data analysis. LB, JR study conception and design. LB, EN, AF, KA, TH, SN, MR, MZ, JR acquisition of data. LB, JR analysis and interpretation of data.
